# The eukaryotic signal sequence, YGRL, targets the chlamydial inclusion

**DOI:** 10.3389/fcimb.2014.00129

**Published:** 2014-09-11

**Authors:** Emily J. Kabeiseman, Kyle H. Cichos, Elizabeth R. Moore

**Affiliations:** Division of Basic Biomedical Sciences, Sanford School of Medicine, University of South DakotaVermillion, SD, USA

**Keywords:** *Chlamydia trachomatis*, chlamydial inclusion, YGRL, trafficking, trans-Golgi, syntaxin 6

## Abstract

Understanding how host proteins are targeted to pathogen-specified organelles, like the chlamydial inclusion, is fundamentally important to understanding the biogenesis of these unique subcellular compartments and how they maintain autonomy within the cell. Syntaxin 6, which localizes to the chlamydial inclusion, contains an YGRL signal sequence. The YGRL functions to return syntaxin 6 to the trans-Golgi from the plasma membrane, and deletion of the YGRL signal sequence from syntaxin 6 also prevents the protein from localizing to the chlamydial inclusion. YGRL is one of three YXXL (YGRL, YQRL, and YKGL) signal sequences which target proteins to the trans-Golgi. We designed various constructs of eukaryotic proteins to test the specificity and propensity of YXXL sequences to target the inclusion. The YGRL signal sequence redirects proteins (e.g., Tgn38, furin, syntaxin 4) that normally do not localize to the chlamydial inclusion. Further, the requirement of the YGRL signal sequence for syntaxin 6 localization to inclusions formed by different species of *Chlamydia* is conserved. These data indicate that there is an inherent property of the chlamydial inclusion, which allows it to recognize the YGRL signal sequence. To examine whether this “inherent property” was protein or lipid in nature, we asked if deletion of the YGRL signal sequence from syntaxin 6 altered the ability of the protein to interact with proteins or lipids. Deletion or alteration of the YGRL from syntaxin 6 does not appreciably impact syntaxin 6-protein interactions, but does decrease syntaxin 6-lipid interactions. Intriguingly, data also demonstrate that YKGL or YQRL can successfully substitute for YGRL in localization of syntaxin 6 to the chlamydial inclusion. Importantly and for the first time, we are establishing that a eukaryotic signal sequence targets the chlamydial inclusion.

## Introduction

Depending on the mode of acquisition, *C. trachomatis* serovars cause several types of infections including blinding trachoma (Schachter, 1999), or the most common bacterially associated sexually transmitted infection (STI). Around 70% of chlamydial infections are asymptomatic and are considered “silent infections,” increasing a patient's risk for developing chronic conditions such as pelvic inflammatory disease, ectopic pregnancy, and infertility. *Chlamydia pneumoniae* is thought to contribute to the two to five million cases of respiratory pneumonia per year, although the actual incidence of *C. pneumoniae*-induced disease is unknown due to poor surveillance/detection in clinical settings (cdc.gov., 2005).

Chlamydiae have evolved a unique biphasic developmental cycle. To initiate an infection, the infectious form, termed the elementary body (EB), is endocytosed into the host cell and remains membrane-bound in a vesicle which develops into a pathogen-specified organelle, known as the inclusion. Within the inclusion, the EB differentiates into a metabolically active, noninfectious reticulate body (RB). The RB actively remodels the surrounding vacuolar membrane with secreted proteins (Fields et al., 2003). These early modifications to the inclusion correspond with the chlamydial acquisition of host-derived lipids (Hackstadt et al., 1996). The intracellular growth and division of *Chlamydia* within the inclusion is essential to the completion of the chlamydial development cycle which culminates in the release of infectious progeny (Ward, 1988). As a pathogen-specified organelle, the chlamydial inclusion membrane provides a defined interface between pathogen and host and a protected environment conducive with chlamydial development, which dictates pathogen fitness. As with other organelles, the function of the inclusion is determined, in part, by the protein and lipid composition of its membrane. This study examines how eukaryotic proteins are targeted to this pathogen-specified organelle.

The inclusion is often conceptualized as a parasitophorous black hole, leeching the host cell of nutrients. But mounting data challenge this notion. For example, there are no gross lipid-associated metabolic changes and host cells remain relatively healthy (Fan and Jenkin, 1974, 1975; Beatty, 2007; Chin et al., 2012). Furthermore, bulk-flow exocytosis is not impeded or interfered with by the chlamydial inclusion (Scidmore et al., 1996). Recently, it was demonstrated that a eukaryotic signal sequence, YGRL, which typically returns proteins to the trans-Golgi from the plasma membrane, also targets eukaryotic ***s***oluble ***N***SF ***a***ttachment protein ***re***ceptor (SNARE) syntaxin 6 to the chlamydial inclusion (Moore et al., 2011). Combined, these data suggest that the inclusion membrane is recognized in a similar way as other organelles and that proteins may cycle between the inclusion and other organelles. Demonstrating that these trafficking dynamics exist is critical to understanding the structure and function of the chlamydial inclusion membrane.

Members of the SNARE family govern the majority of membrane fusion events in the eukaryotic cell (Sollner et al., 1993). SNARE proteins act to overcome the energy requirements of membrane fusion events, and specific SNARE complexes function at distinct organelles within the cell (Jahn and Scheller, 2006; Bethani et al., 2007; Lam et al., 2008). Regarding the localization of SNAREs to the chlamydial inclusion, we have demonstrated that syntaxin 6, but not syntaxins 4 and 16 localize to the inclusion. The localization of syntaxin 6 requires chlamydial protein synthesis and is conserved across species, suggesting that syntaxin 6 significantly contributes to the chlamydial intracellular lifestyle (Moore et al., 2011). Further, we have demonstrated that syntaxin 6 and VAMP4 localize to and interact at the inclusion (Kabeiseman et al., 2013). Although the syntaxin 6-VAMP4 interaction is not conserved across chlamydial species, the interaction is a strong indicator that SNARE complexes are forming at the chlamydial inclusion. How these SNARE proteins are targeted and trafficked to the inclusion is inextricably linked to function.

We have previously demonstrated that the YGRL signal sequence within syntaxin 6 is required for its localization to the chlamydial inclusion (Moore et al., 2011). The YGRL signal sequence is a member of a larger collection of YXXL signal sequences (YQRL and YKGL), which function to recycle proteins to the trans-Golgi region along basolateral trafficking pathways (Bos et al., 1993; Humphrey et al., 1993; Wong and Hong, 1993; Molloy et al., 1994; Rajasekaran et al., 1994; Reaves et al., 1998; Simmen et al., 1999). Given that the chlamydial inclusion intercepts basolateral exocytic vesicles (Moore et al., 2008), we wanted to understand if other YXXL signal sequences recognize the chlamydial inclusion, if the YGRL signal sequence recognized chlamydial inclusions formed by other chlamydial species, and if this signal sequence modulated protein-protein or protein-lipid interactions. Importantly, we are establishing that the YGRL signal sequence can target other proteins (not just syntaxin 6) to the chlamydial inclusion. Significantly, we are examining mechanisms of how proteins are targeted to a pathogen-specified organelle and are demonstrating, for the first time, that a eukaryotic signal sequence specifically targets the chlamydial inclusion.

## Materials and methods

### Organisms and cell culture

HeLa 229 cells [American Type Culture Collection (ATCC); Manassas, VA; CCL-2.1] were cultured at 37°C in 5% CO_2_ with RPMI 1640 or DMEM (HyClone/Thermo Scientific, Logan, UT), supplemented with 10% fetal bovine serum (FBS) (HyClone) and 10 μg/ml gentamicin (Gibco-BRL/Life Technologies; Grand Island, NY). HeLa cells were used to propagate *Chlamydia trachomatis* serovar L2 (LGV 434) for purification using established protocols (Caldwell et al., 1981; Scidmore, 2005). Chlamydial titers were determined using conventional protocols to establish multiplicities of infection (m.o.i.), which are based on inclusion forming units (i.f.u.) and determined in HeLa cells, essentially as previously described (Furness et al., 1960; Scidmore, 2005).

### Creation of 3XFLAG and BioID constructs

All primers used in these cloning projects are listed in Table 1 (Supplementary Material). To generate amino-terminal 3XFLAG fusion constructs, PCR was used to introduce appropriate 5′ and 3′ restriction sites to furin, syntaxin 4, and Tgn38 cDNA (Origene, Rockville, MD). The PCR products were cloned in frame to the 3XFLAG coding sequence in the p3XFLAG-CMV 7.1 expression vector (Sigma Aldrich, St. Louis, MO). The GeneTailor Site-Directed Mutagenesis system (Life Technologies) was used to create 3XFLAG-syntaxin 4+YGRL, 3XFLAG-syntaxin 4 +YKGL, and 3XFLAG-syntaxin 4 +YQRL and 3XFLAG-TGN38 _YGRL, 3XFLAG-TGN38ΔYQRL, and 3XFLAG-furinΔYKGL constructs using previously described conditions (Moore et al., 2011). The 3XFLAG-furin_YGRL mutant was constructed by three-step site-specific PCR mutagenesis (Shokeen et al., 2008) using end, mutagenic and overlapping primers listed in Table 1. The overlap extension PCR cloning method (Bryksin and Matsumura, 2010) was used to create the 3XFLAG-syntaxin 6 and 3XFLAG-syntaxin 6 ΔYGRL (both constructs previously described in Moore et al., 2011) into the pcDNA3.1mycBioID plasmid (plasmid # 35700, Addgene.org) (Roux et al., 2012). All constructs were confirmed by sequencing (Eurofins MWG Operon, Hunstville, AL).

**Table 1:** **Primers used in creation of 3XFLAG and BioID constructs**.

**Primer**	**Sequence**	**Purpose**
1^a^	5′-GGGGAATTCAATGCGGTTCGTAGTTGCC-3′	Cloning Tgn38 into 3XFLAG vector
2	5′-GGGGGATCCTCACTTCTGGTCCAAACGTTG-3′	
3	5′-CGTTTGGACCAGAAGTGAGGATCCCG-3′	3XFLAG Tgn38 Q431G mutation
4	5′-ACTTCTGGTCCAAACG*gcc*GTAGTCACTG-3′	
5	5′-GGGGGAAGCTTATGCGGGACAGGACCCAC-3′	Cloning syntaxin 4 into 3XFLAG vector
6	5′-GGGGGGAATTCTTATCCAACCACTGTGACGC-3′	
7	5′-AGCAGATGCTGGACAGTGGG*tatgggcgcctt*CAAAGCGAGG-3′	Adding YGRL to 3XFLAG syntaxin 4
8	5′-CCCACTGTCCAGCATCTGCTCCAACTCCTC-3′	
9	5′-GCAGATGCTGGACAGTGGG*taccaacgtttg*CAAAGCGAGG-3′	Adding YQRL to 3XFLAG syntaxin 4
10	5′-CCCACTGTCCAGCATCTGCTCCAACTCCT-3′	
11	5′-CAGATGCTGGACAGTGGG*tacaaggggctg*CAAAGCGAGG-3′	Adding YKGL to 3XFLAG syntaxin 4
12	5′-CCCACTGTCCAGCATCTGCTCCAACTCCTC-3′	
13	5′-GGGGGGGAATTCTCAGAGGGCGCTCTGGTC-3′	Cloning furin into 3XFLAG vector; end primers for site specific PCR mutagenesis
14	5′-GGGGGGAAGCTTGACGTGTACCAGGAG-3′	
15	5′-CTCATCTCCTACGGGCGGCTGC-3′	Mutagenic and overlapping primers for site specific PCR mutagenesis of furin K677G and G678R
16	5′-GCAGCCGCCCGTAGG*a*GATGAG-3′	
17	5′-GAGAAATCTCCCTGAGAAGCGCAGAGAAGATGGACTACAAAGACCATGACG-3′	Cloning stx6 and stx6ΔYGRL into BioID using overlap extension PCR
18	5′-GCAACTAGAAGGCACAGTCGAGGCTGATCACAGCACTAGGAAGAGGGT-3′	
21	5′-GGCCAAAGGCCAGTGA***CG***ACCAGAAGTGA-3′	Deleting YQRL from Tgn38
22	5′-GTCACTGGCCTTTGGCCGCCGGGTGAC-3′	
23	5′-TGGACCGTGGCCTCATCTC***CC***CCCCTGAAG-3′	Deleting YKGL from furin
24	5′-GGAGATGAGGCCACGGTCCATGGTGTACAC-3′	

a *All odd numbered primers are forward primers and all even numbered primers are reverse primers*.

*Restriction sites are underlined. Mutated sequences are in lower case and italicized. Deletions are indicated with bold and italics on either side of the deleted sequence. For overlap extension PCR vector sequence is underlined*.

### Biotinylation studies

100 ng of BioID DNA constructs were transfected into HeLa cells as described previously (Kabeiseman et al., 2013). HeLa cells were grown in biotin-free media (DMEM + 10% FBS) prior to transfection. After transfection, cells were grown in media supplemented with 50 μM biotin for 2 h, and then infected with *C. trachomatis* (m.o.i. 1) for 16 h. The cells were fixed for 10 min in 4% paraformaldehyde and permeablized for 5 min in 0.1% saponin or 0.5% Triton-X100 (Sigma Aldrich). Coverslips were processed for indirect immunofluorescence to detect the BioID constructs with mouse anti-FLAG M2 (Sigma Aldrich), followed by the appropriate DyLight fluor conjugated secondary antibody (Jackson ImmunoResearch Laboratories, West Grove, PA). Biotinylated proteins were detected with streptavidin conjugated to Alexa Fluor 488 (Pierce/Thermo, Rockford, IL). The cells were also stained with DAPI to visualize chlamydial organisms and nuclei. Coverslips were mounted onto slides using Prolong Gold antifade mounting media (Life Technologies) and visualized with an Olympus BX60 fluorescent scope (60× magnification). Images were taken with a Nikon DS-Qi1Mc camera.

### 3XFLAG immunofluorescence

HeLa cells were transfected with 100 ng of plasmid as described previously (Kabeiseman et al., 2013). Transfected cells were infected with either *C. trachomatis* serovar L2 (m.o.i. 1) or *Chlamydia muridarum* (m.o.i. 0.1) for 16 h prior to fixation in ethanol for 20 min at −20°C. HeLa cells were infected with *Chlamydia pneumonia* (m.o.i. 4) or *C. burnetii* nine mile phase II (m.o.i. 42) for 48 and 72 h prior to transfection, respectively. The coverslips were processed for indirect immunofluorescence using mouse anti-FLAG M2, rabbit anti-IncG (kindly provided by Ted Hackstadt, NIAID, Rocky Mountain Laboratories, Hamilton, MT), rabbit anti-14-3-3β (Santa Cruz Biotechnology, Dallas, Texas), guinea pig anti-*C. burnetii* and DAPI. The appropriate secondary antibodies were all conjugated to DyLight fluors (Jackson ImmunoResearch Laboratories). Coverslips were mounted and imaged either as described above or with the Olympus Fluoview 1000 Laser Scanning Confocal Microscope.

### 3XFLAG immunoprecipitation

HeLa cells were seeded into a 10 cm dish and allowed to grow overnight. The cells were transfected with 1.0 μg of the 3XFLAG vector, 3XFLAG-syntaxin 6 or 3XFLAG-syntaxin 6 ΔYGRL constructs, infected and 3XFLAG-fusion proteins were purified, essentially as previously described (Kabeiseman et al., 2013), with the exception that of the cell lysis buffer [50 mM TricHCl, pH 7.4, 150 mM NaCl, 1%NP-40, 1X HALT protease inhibitor cocktail (Pierce/Thermo), and 150 μM clastolactacystin (Caymen Chemicals, Ann Arbor, MI). Immunoprecipitated proteins were eluted in sample buffer. Samples were analyzed by Western blotting with mouse anti-FLAG M2 to detect 3XFLAG-syntaxin 6 and ΔYGRL], rabbit anti-VAMP4 (Sigma Aldrich), rabbit anti-syntaxin 5 (Synaptic Systems, Goettingen, Germany), mouse anti-Vti1a [BD Biosciences, San Jose, CA)] and mouse anti-GAPDH (EMD Millipore, Billerica, MA) and secondary antibodies goat anti-rabbit IRDye 800CW- and goat anti-mouse IRDye 680LT-conjugated antibodies (LiCor Biosciences, Lincoln, NE). Images were taken using the Odyssey CLx and processed using Image Studio version 2.0 (LiCor Biosciences). Additionally samples were analyzed by staining 4–20% gradient SDS-PAGE gels with SYPRO Ruby (BioRad, Hercules, CA) staining as described by the manufacturer and images were taken using the Typhoon imager (GE Healthcare Bio-Sciences). Protein bands of interest were excised with a robotic spot cutter (BioRad), in-gel digested using sequencing grade trypsin (Promega, Madison, WI) and desalted using an in-line nanoRPLC as described previously (Mo et al., 2006, 2008). Peptide analysis was performed using nanoLC-ESI-MS/MS [nanoAcquity UPLC coupled to Q-Tof Synapt G1 MS (Waters, Milford, MA)]. More details provided in Supplementary Materials.

### Protein-lipid interactions assays

Immunopreciptated 3XFLAG-syntaxin 6 and 3XFLAG-syntaxin 6 ΔYGRL were incubated with Sphingostrips (S-6000) or PIPstrips (P-6001) (Echelon Biosciences Inc., Salt Lake City, UT). Briefly, 3XFLAG-syntaxin 6 and 3XFLAG-syntaxin 6 ΔYGRL were immunopreciptated from HeLa cells as described above. Protein eluates were quantitated and 8.4 or 2.5 μg of protein was incubated for 24 h in Tris buffered saline (TBS) (50 mM Tris, pH 7.4, and 150 mM NaCl) with Sphingostrips or PIPstrips, respectively. Unbound proteins were removed by washing three times with TBS-0.1%Tween-20. The strips were then processed by blotting with mouse anti-FLAG M2 primary antibody and goat anti-mouse IRDye 680LT-conjugated secondary antibody in TBS. Images were taken using the Odyssey CLx and processed with Image Studio version 2.0 (LiCor Biosciences). Protein input for sphingostrips and PIPstrips was analyzed by SDS-PAGE and Western blot as described above. Fold increase in syntaxin 6-lipid binding was determined by normalization of densitometry as described below.

### Densitometry and image production

All Western blots (including Spingostrips and PIPstrips) were analyzed for densitometry using Image Studio Version 2.0 software (LiCor Biosciences). Raw densitometry units were normalized in Excel (Supplemental Material). Graphed data (averages and standard deviation or standard error of the mean) was produced using GraphPad Prism 6 software (GraphPad Software, La Jolla, CA). All figures were constructed using Adobe Photoshop CS5 (Adobe Systems Incorporated, San Jose, CA). Any modifications to images were limited to adjustment of color balance in fluorescent images, or brightness and contrast in Western blot images.

## Results

### Deletion of YGRL prevents syntaxin 6 from associating with the inclusion

**Figure 1:**
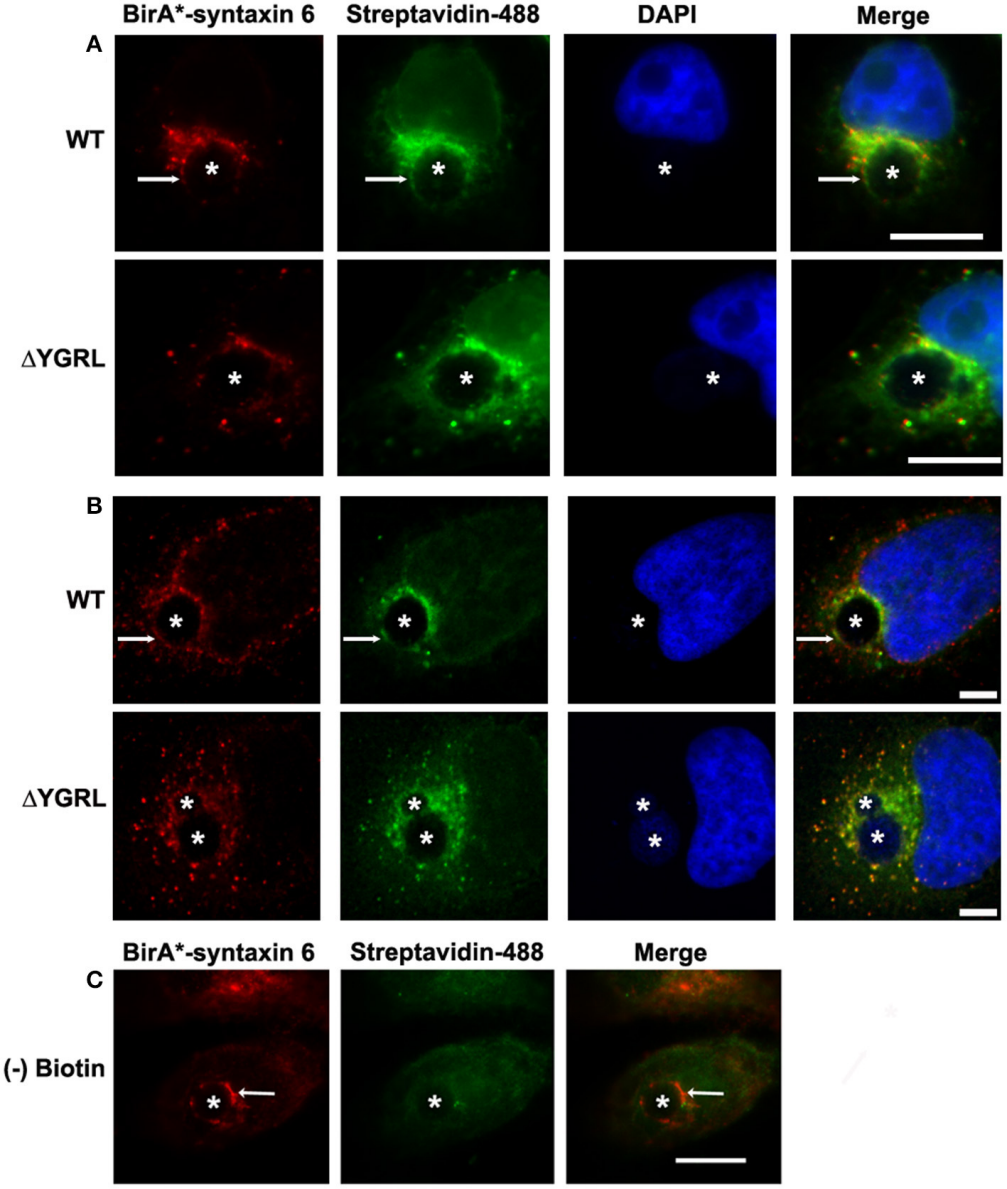
**Biotinylation of the chlamydial inclusion**. HeLa cells were transfected with BirA^*^ syntaxin 6 WT (WT) or BirA^*^-syntaxin 6 ΔYGRL (ΔYGRL) and infected with *C. trachomatis* serovar L2. Cells were also treated with **(A,B)** or without **(C)** exogenous biotin. Then, cells were fixed and processed for indirect immunofluorescence assay to detect the BirA^*^ construct (red) and biotin (green) and imaged with either a Olympus BX60 fluorescent scope (60× magnification) **(A,C)** or an Olympus Fluoview 1000 Laser Scanning Confocal Microscope **(B)**. The cells were stained with DAPI to detect the chlamydial inclusion and nuclei (blue). Each image is representative of at least two independent experiments. White stars designate the chlamydial inclusion and white arrows denote colocalization of syntaxin 6 WT with the chlamydial inclusion. Bars, 10 μm **(A,C)** or 5 μm **(B)**.

In a previous study, deletion of the YGRL signal sequence from syntaxin 6 (syntaxin 6ΔYGRL) resulted in the protein localizing around but not with the chlamydial inclusion (Moore et al., 2011). To confirm that syntaxin 6ΔYGRL does not associate or interact, even transiently, with the chlamydial inclusion, we utilized a new technique called BioID (Roux et al., 2012). The BioID method utilizes a mammalian expression vector (pcDNA3.1mycBioID), which encodes a promiscuous biotin ligase (BirA^*^) to which *syntaxin 6* or *syntaxin 6ΔYGRL* is cloned to create fusion constructs: *BirA-syntaxin 6* or *BirA-syntaxin 6ΔYGRL*. Proteins within 20 nm of the BirA^*^-fusion protein will be biotinylated by BirA^*^ when cultured with exogenous biotin (Roux et al., 2012). The exogenous biotin is added to the culture for 24 h prior to fixation, so the final result is a record of *all* of the subcellular localizations that the protein has been trafficked, in addition to the subcellular location of the protein at the time of fixation. Hence, we are asking the important question of whether syntaxin 6 is trafficked to, and then retained, at the chlamydial inclusion. BirA^*^-syntaxin 6 localizes to the chlamydial inclusion and biotinylates the chlamydial inclusion membrane, as well as associated Golgi structures (Figures 1A,B). In contrast, but consistent with previous studies (Moore et al., 2011), BirA^*^-syntaxin 6ΔYGRL is not trafficked to the chlamydial inclusion and the chlamydial inclusion is not biotinylated; however, surrounding vesicular structures are biotinylated (Figures 1A,B). Recent studies demonstrated that only a single protein within *Chlamydia* is biotinylated (Fisher et al., 2012), which is consistent with the lack of structures being biotinylated by BirA^*^-syntaxin 6 in the absence of exogenous biotin (Figure 1C). Therefore, these data conclusively confirm that the YGRL signal sequence is an absolute requirement for syntaxin 6 to be trafficked to the chlamydial inclusion. Further, lack of biotinylation of the inclusion membrane from the BirA^*^-syntaxin 6ΔYGRL demonstrates conclusively that this construct is never trafficked to the chlamydial inclusion membrane.

### The propensity of YXXL signal sequences to target proteins to the chlamydial inclusion

The YGRL sequence is found within the C-terminal region of syntaxin 6 and is proximal to the soluble *N*-ethylmaleimide sensitive factor attachment protein receptor (SNARE) domain (Watson and Pessin, 2000; Wendler and Tooze, 2001). While syntaxin 6 is predominately found within the trans-Golgi region, the YGRL signal sequence was characterized as a *plasma membrane retrieval signal* as it is required for proper cycling of syntaxin 6 from the plasma membrane to the trans-Golgi region (Watson and Pessin, 2000). The YGRL signal sequence is one of three similar tyrosine-based YXXL signal sequences: YGRL, YQRL and YKGL, which function by returning proteins trafficked to the plasma membrane back to the trans-Golgi. The YKGL signal sequence is required for proper localization of furin (Molloy et al., 1994), and the YQRL signal sequence is required for proper localization of Tgn38 (Bos et al., 1993; Humphrey et al., 1993; Wong and Hong, 1993). To examine if YQRL and YKGL signal sequences targeted the chlamydial inclusion, we examined prototypical proteins furin (YKGL) and Tgn38 (YQRL).

#### Localization of prototypical YXXL-signal sequence containing proteins to the chlamydial inclusion

**Figure 2:**
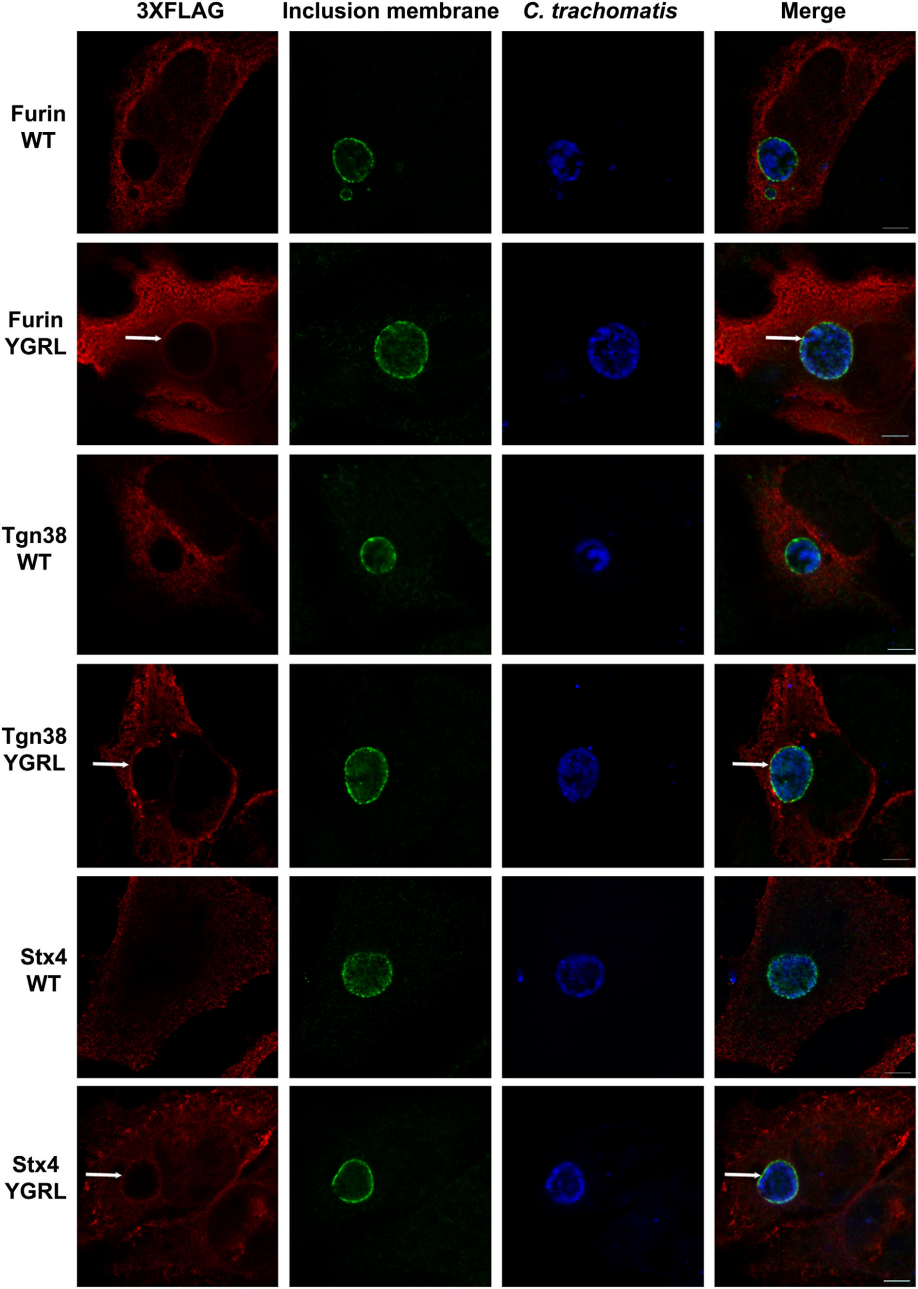
**Addition of the YGRL signal sequence targets furin, tgn38, and syntaxin 4 to the chlamydial inclusion**. HeLa cells were transfected with control construct 3XFLAG vector (C) and 3XFLAG-furin, 3XFLAG-Tgn38, 3XFLAG-syntaxin 4 (Stx4) wild type (WT) and their respective YGRL mutant constructs. After infection with *C. trachomatis* serovar L2 for 18 h, the cells were fixed in ethanol and processed for indirect immunofluorescence assay to detect 3XFLAG (red), the inclusion membrane (IncG, green), *C. trachomatis* (blue). Images were visualized with an Olympus Fluoview 1000 Laser Scanning Confocal Microscope. Each image is representative of at least three independent experiments. White arrows denote colocalization with the chlamydial inclusion. Bars, 5 μm.

To test the propensity of these signal sequences to traffic proteins to the chlamydial inclusion, we have cloned these representative proteins into the p3XFLAG-CMV7.1 vector with their wild type YXXL signal sequence or the alternative YGRL signal sequence. These constructs were transfected into HeLa cells, then infected with *C. trachomatis*. By indirect immunofluorescent microscopy, we demonstrate that neither wild type protein colocalizes with the chlamydial inclusion (Figure 2 and Supplemental Figure 1A). These results are consistent with previous studies that also demonstrated that Tgn38 does not colocalize with the chlamydial inclusion (Vanooij et al., 1997). In contrast, mutation of each YXXL signal sequence to YGRL altered the localization of both 3XFLAG-furin and 3XFLAG-Tgn38 (Figure 2). 3XFLAG-furin_YGRL localized in a perfect rim around the inclusion as syntaxin 6 does (Moore et al., 2011). While, the localization of 3XFLAG-Tgn38_YGRL resembles punctate patches around the chlamydial inclusion (Figure 2 and Supplemental Figure 1A). These data demonstrate that mutation of existing YXXL sequences to YGRL allows the protein to be targeted to the chlamydial inclusion. Further, deletion of YXXL signal sequences from furin or Tgn38 resulted in diffuse localization throughout the cytosol, but no localization to the chlamydial inclusion (Supplemental Figure 2). As a control, localization of 3XFLAG-Tgn38_YGRL (Supplemental Figure 1B) or 3XFLAG-furin_YGRL (Supplemental Figure 3) to avirulent *Coxiella burnetii*-containing parasitophorous vacuole was tested. *C. burnetii* form vacuoles of similar size to *C. trachomatis*-containing inclusions, but interact with different intracellular trafficking pathways (Heinzen et al., 1996). Mutation of the YXXL signal sequence to YGRL did not target 3XFLAG-furin_YGRL or 3XFLAG-Tgn38_YGRL to the *Coxiella* parasitophorous vacuole. Hence, the YGRL-targeting of proteins to the chlamydial inclusion is specific to pathogen specified organelles formed by *Chlamydia*.

#### Effect of addition of YXXL signal sequences to SNARE protein syntaxin 4

**Figure 3:**
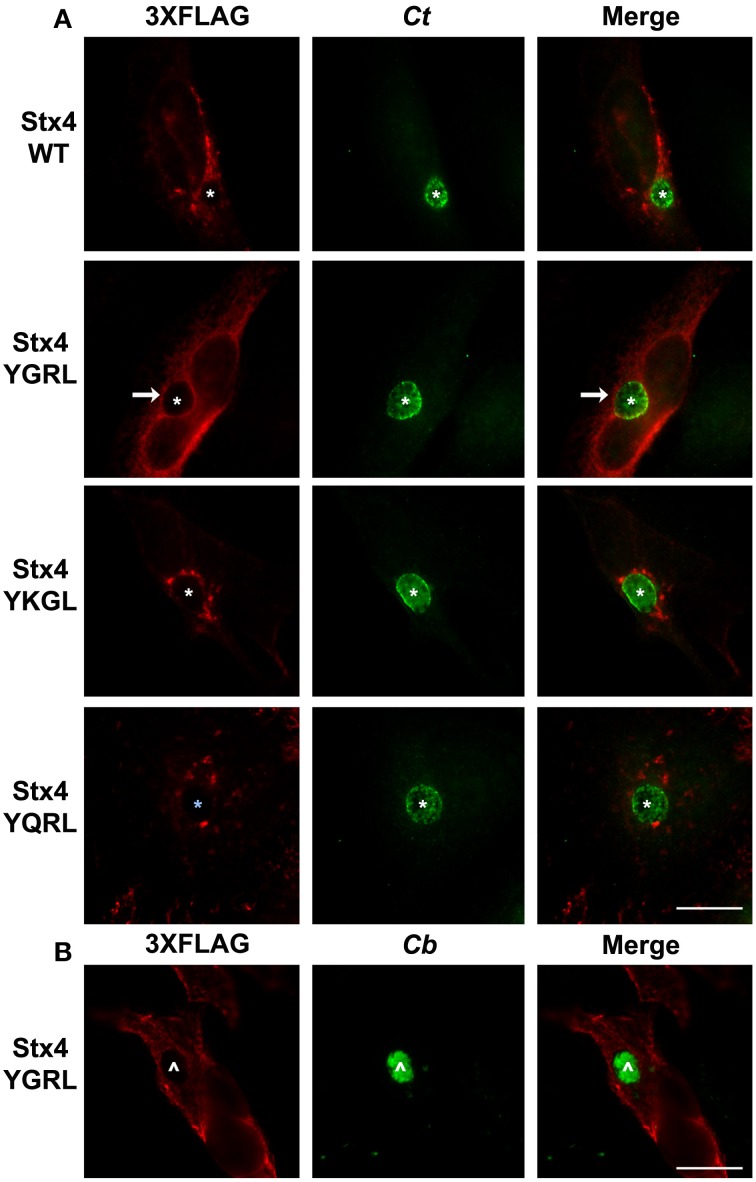
**Examination of syntaxin 4 YXXL mutant colocalization with the chlamydial inclusion**. HeLa cells were transfected with 3XFLAG-syntaxin 4 (Stx4) or one of three 3xFLAG-syntaxin 4 signal sequence insertion mutants and infected with *C. trachomatis* serovar L2 (*Ct*) **(A)** or *C. burnetii* (*Cb*) **(B)**. Cells were fixed in ethanol and processed for indirect immunofluorescence assay to detect 3XFLAG (red), *C. trachomatis* [green, **(A)**] or *C. burnetii* [green, **(B)**]. Each image is representative of at least two independent experiments. White stars designate the chlamydial inclusion, white caret designates the *C. burnetii* vacuole and white arrows denote colocalization of syntaxin 4-YGRL with the chlamydial inclusion. Bars, 10 μm.

Furin and Tgn38 have fundamentally different protein structures/organization from syntaxin 6, and these structures ultimately impact their subcellular localization and protein-binding partners. Both furin and Tgn38 have N-terminal regions that remain with the vesicle lumen, a single membrane-spanning domain, and a C-terminal cytosolic tail, which contains the YXXL signal sequence (Bos et al., 1993; Humphrey et al., 1993; Wong and Hong, 1993; Jones et al., 1995). In contrast, syntaxin 6 is anchored to membranes with a C-terminal transmembrane domain and localizes on the cytosolic face of vesicles with the YGRL signal sequence located proximal to the SNARE domain (Watson and Pessin, 2000; Wendler and Tooze, 2001). Syntaxin 4 is a protein similar in structure to syntaxin 6, yet it does not localize to the chlamydial inclusion (Weimbs et al., 1997; Fasshauer et al., 1998; Moore et al., 2011). Syntaxin 4 also does not contain an YXXL signal sequence (Bennett et al., 1993). As it is not practical to screen the localization of all proteins containing a YGRL, YQRL or YKGL signal sequence for their localization to the chlamydial inclusion, we tested the ability of the YXXL signal sequences to target a protein to the chlamydial inclusion by adding YGRL, YQRL or YKGL to syntaxin 4 in approximately the same position as the YGRL is located in syntaxin 6. HeLa cells were transfected with the indicated constructs and then infected with *C. trachomatis*. Wild type 3XFLAG-syntaxin 4 localizes along the plasma membrane and within vesicles in the cell, but does not localize to the chlamydial inclusion (Figures 2, 3A), as previously demonstrated (Moore et al., 2011). Syntaxin 4 with YXXL signal sequences localizes in the Golgi region and in vesicles throughout the cell. Notably only 3XFLAG-syntaxin 4+YGRL localizes to the chlamydial inclusion (Figures 2, 3A). 3XFLAG-syntaxin 4+YGRL does not localize to the *Coxiella*-parasitophorous vacuole (Figure 3B). In summary, addition of YGRL to syntaxin 4, which normally does not localize to the chlamydial inclusion, targets this protein to the chlamydial inclusion. Further, the propensity of the YGRL signal sequence to target the inclusion is specific for the chlamydial inclusion.

#### The YGRL signal sequence targets inclusions of multiple chlamydial species

**Figure 4:**
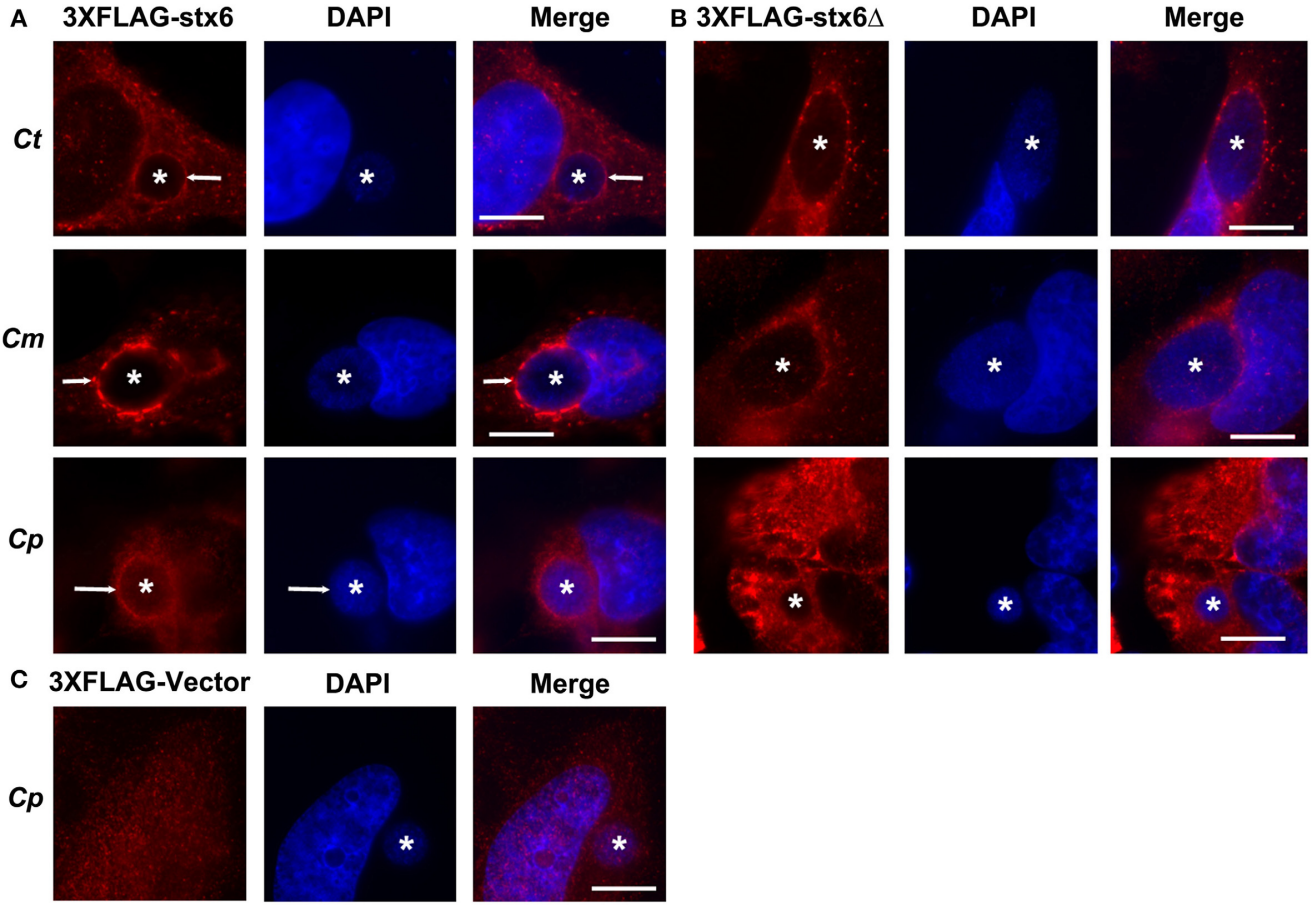
**Localization of syntaxin 6 and syntaxin 6ΔYGRL to different chlamydial species**. HeLa cells were transfected with 3XFLAG empty vector (3XFLAG-Vector) **(C)**, 3XFLAG-syntaxin 6 (3XFLAG-Stx6) **(A)**, or 3XFLAG-syntaxin 6ΔYGRL (3XFLAG-Stx6Δ) **(B)** and infected with *C. trachomatis* serovar L2 (*Ct*), *C. muridarum* (*Cm*), or *C. pneumoniae* (*Cp*). The cells were fixed in ethanol after 24 h (*Ct* and *Cm*) or 72 h (*Cp*) of infection and processed for indirect immunofluorescence assay to detect 3XFLAG constructs (red). The cells were stained with DAPI to detect the chlamydial inclusion and nuclei (blue). Each image is representative of at least two independent experiments. White stars designate the chlamydial inclusion and white arrow denotes colocalization of syntaxin 6 with the chlamydial inclusions. Bars, 10 μm.

Previous studies established that localization of syntaxin 6 to the chlamydial inclusion is conserved across chlamydial species (Moore et al., 2011). Hence, we examined if the requirement of the YGRL signal sequence for syntaxin 6 localization to the inclusion was also conserved across chlamydial species. By indirect immunofluorescence, we examined 3XFLAG-syntaxin 6 and 3XFLAG-syntaxin 6ΔYGRL localization to inclusions formed by *C. muridarum* and *C. pneumoniae* in comparison with the localization observed to inclusions formed by *C. trachomatis* serovar L2. Consistent with previous studies (Moore et al., 2011), 3XFLAG-syntaxin 6 formed distinct rims around inclusions of multiple chlamydial species (Figure 4A). Deletion of the YGRL resulted in no localization of 3XFLAG-syntaxin 6 to inclusions formed by any serovars/species (Figure 4B). Additionally, the 3XFLAG construct alone (empty vector) did not target the chlamydial inclusion (shown *C. pneumoniae*, Figure 4C). These data demonstrate that the YGRL recognizes inclusions formed by multiple chlamydial species.

### Role of YGRL in syntaxin 6 protein-protein and protein-lipid interactions

We have demonstrated that deletion of the YGRL prevents the trafficking of syntaxin 6 to the chlamydial inclusion. To understand if syntaxin 6 protein-protein or protein-lipid interactions mediate the YGRL recognition of the inclusion membrane, we asked if the deletion of the YGRL altered syntaxin 6-protein interactions or syntaxin 6-lipid interactions.

#### Effect of the YGRL on syntaxin 6 protein-protein interactions

**Figure 5:**
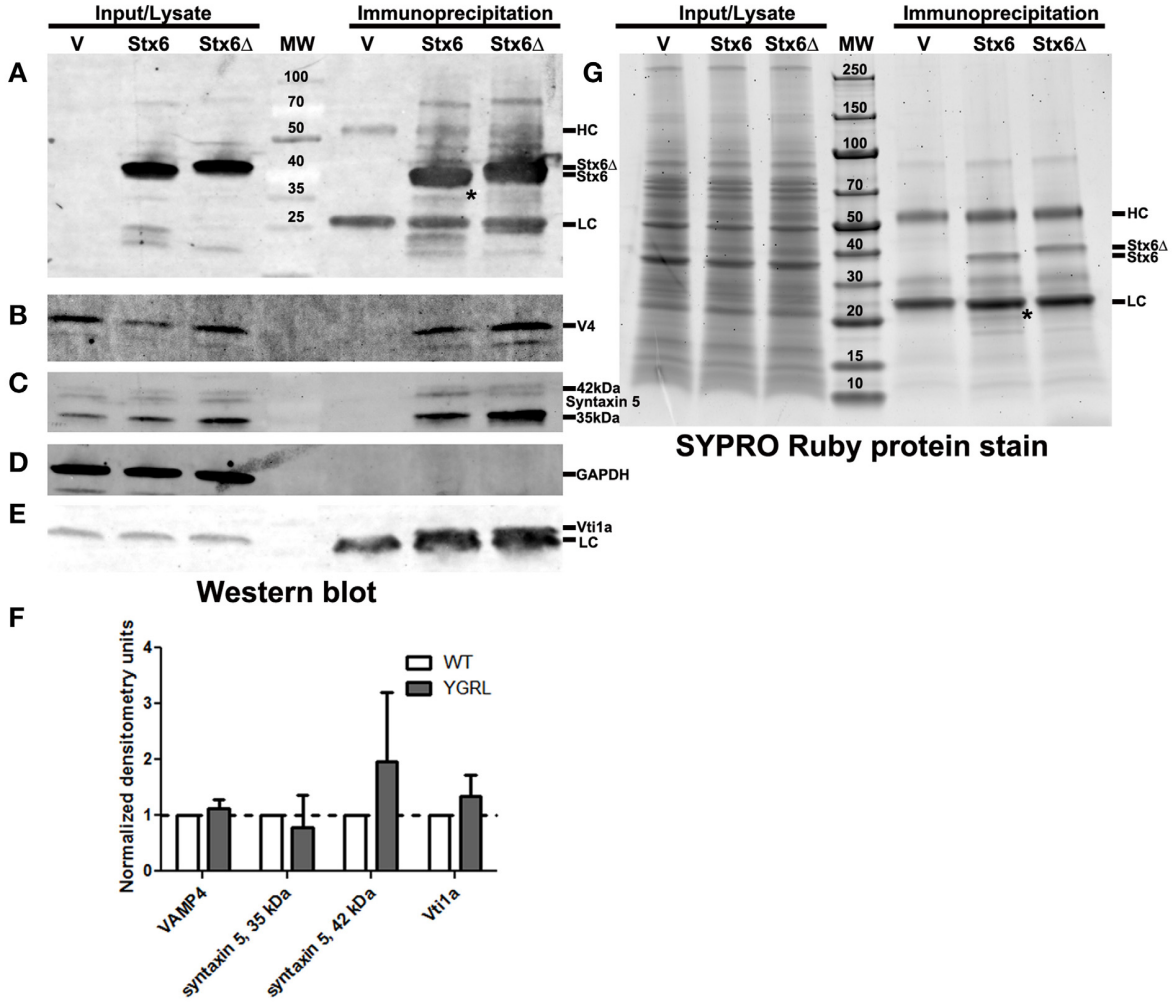
**Immunoprecipitation analysis of syntaxin 6 and syntaxin 6ΔYGRL**. Lysates from transfected and *C. trachomatis* infected HeLa cells were immunoprecipitated with anti-FLAG M2 antibody. The input and immunoprecipitates were resolved by SDS-12%PAGE and blotted with antibodies against FLAG M2 **(A)**, VAMP4 (V4) **(B)**, syntaxin 5 **(C)**, GAPDH **(D)**, and Vti1a **(E)**. Densitometry values of eluates of each protein were normalized to input bands and the amount of 3XFLAG-syntaxin 6 (Stx6) or 3XFLAG-syntaxin 6ΔYGRL (Stx6Δ) eluates. The normalized values are expressed as a ratio to Stx6 eluates (set at 1) and the resulting graph (average and standard deviation as calculated by GraphPad Prism software) is representative of values obtained in two independent experiments **(F)**. Additionally, the input and immunoprecipitates were resolved with gradient SDS-4–20%PAGE gels and stained overnight with SYPRO Ruby protein stain **(G)**. ^*^Denotes bands which were similar in size to bands on the Western blots and SYPRO Ruby protein stains. The location of the heavy chain (HC), light chain (LC), 3XFLAG-syntaxin 6, and 3XFLAG-syntaxin 6 ΔYGRL are indicated in panels **(A,G)**. The images are representative of at least two independent experiments.

Cells were transfected with 3XFLAG-syntaxin 6 or 3XFLAG-syntaxin 6ΔYGRL, then infected with *C. trachomatis* serovar L2 for 24 h. Cells were lysed and processed for immunoprecipitation using the 3XFLAG epitope affinity for M2 Agarose as described in *Materials and Methods*. Samples were resolved by SDS-PAGE, then processed for Western blot or SYPRO Ruby protein stain. Western blot against the FLAG epitope revealed similar levels of expression of the 3XFLAG-syntaxin 6 and 3XFLAG-syntaxin 6ΔYGRL in cells (*input*) and similar levels of protein were immunoprecipitated (*immunoprecipitation*) from cells (Figure 5A). We also tested the ability of 3XFLAG-syntaxin 6 and 3XFLAG-syntaxin 6ΔYGRL to bind to known binding partners VAMP4 (Steegmaier et al., 1999; Mallard et al., 2002), Vti1a (Kreykenbohm et al., 2002), and syntaxin 5 (Laufman et al., 2013). To ensure that our immunoprecipitation protocol did not lend itself to nonspecific binding, we used GAPDH as a negative control, as it does not interact with syntaxin 6 within the cell (Figure 5D). Of the known binding partners, VAMP4 interacts with syntaxin 6 at the chlamydial inclusion (Kabeiseman et al., 2013), while Vti1a and syntaxin 5 do not localize to the chlamydial inclusion (Supplementary Figure 4). Deletion of YGRL from syntaxin 6 did not inhibit syntaxin 6 from binding to any of the protein binding partners examined (Figures 5B,C,E). Notably, deletion of the YGRL results in an increase of binding between syntaxin 6 and the 42 kDa isoform of syntaxin 5 and slightly enhances binding of syntaxin 6 with Vti1a (Figures 5B,E).

To examine syntaxin 6 protein-protein interactions more broadly, we resolved these samples by SDS-PAGE followed by SYPRO Ruby protein stain. The only changes that we observed between the wild type and ΔYGRL syntaxin 6 were that 3XFLAG-syntaxin 6 interacts with a unique ~20 kDa protein, and exhibited a stronger interaction with an ~18 kDa protein (Figure 5G). Upon MS-MS identification of the proteins, both proteins are concluded to be breakdown products of the 3XFLAG-syntaxin 6 construct itself (based on peptide sequence AVNTAQGLFQR with Mascot score of 84.9, matching syntaxin 6-isoform 1, *Homo sapiens*), and is consistent with Western blots of these samples with the anti-FLAG M2 antibody (Figure 5A). Therefore, we conclude that the deletion of the YGRL signal sequence of syntaxin 6 does not inhibit syntaxin 6 from interacting with known binding partners.

#### Effect of the YGRL on syntaxin 6 protein-lipid interactions

**Figure 6:**
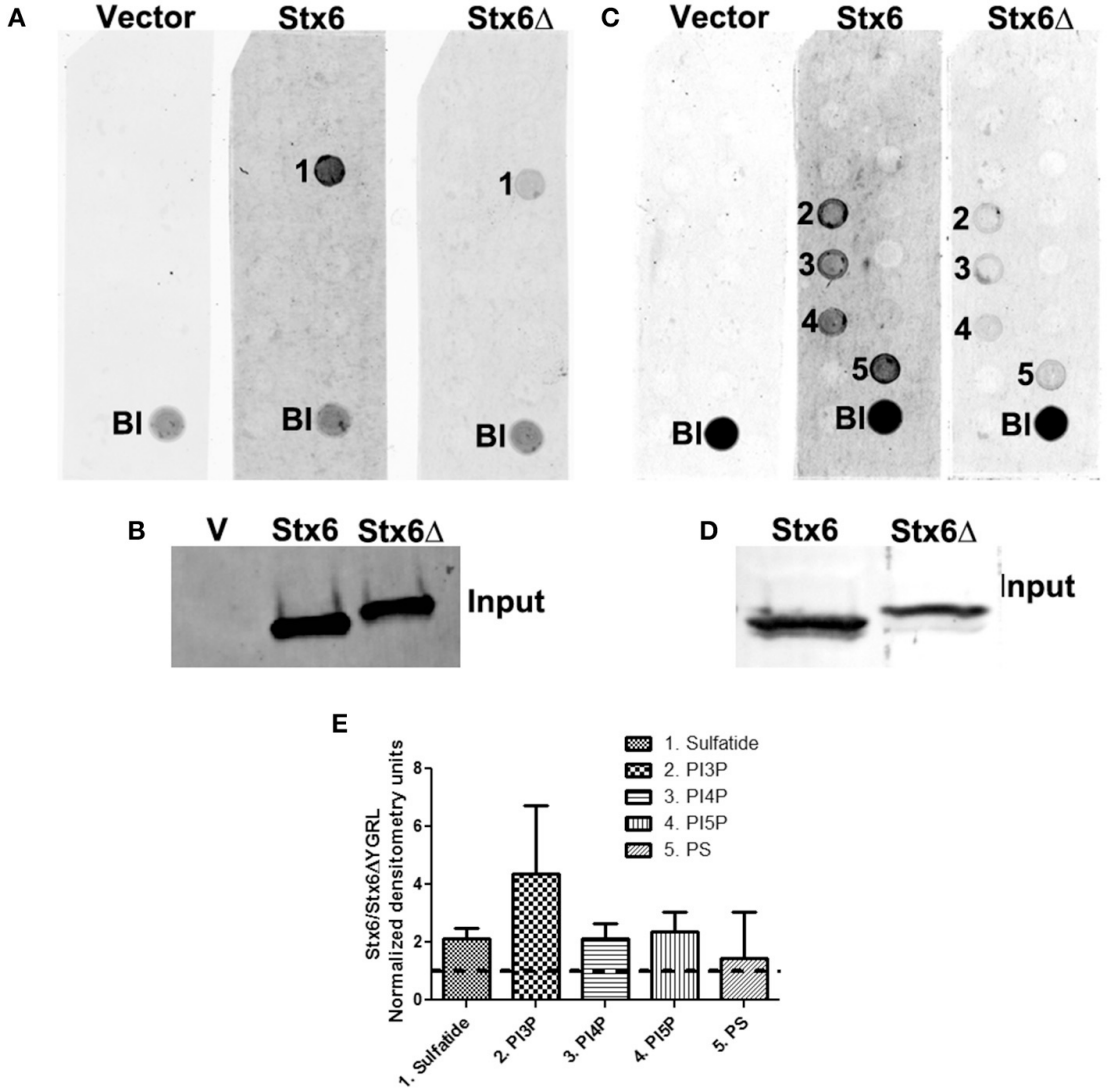
**Syntaxin 6 and syntaxin 6ΔYGRL protein-lipid interactions**. 3XFLAG empty vector (V), 3XFLAG-syntaxin 6 (Stx6) and 3XFLAG-syntaxin 6ΔYGRL (Stx6Δ) were immunopreciptated from HeLa cells and incubated with Sphingostrips **(A)** or PIPstrips **(C)**. Protein-lipid interactions with sulfatide (1), phosphatidylinositol 3-phosphate (PI3P) (2), phosphatidylinositol 4-phosphate (PI4P) (3), phosphatidylinositol 5-phosphate (PI5P) (4), and phosphatidylserine (PS) (5) were detected by blotting with anti-3XFLAG. The blank (Bl) is illuminated for orientation purposes. Protein input for sphingostrips and PIP strips was analyzed by blotting with anti-FLAG M2 antibody (**B,D**, respectively). Fold increase in syntaxin 6-lipid binding was determined by normalization of densitometry to input **(E)**. Graph depicts the fold changes in densitometry levels of Stx6 compared to Stx6ΔYGRL. Positive reactions on Sphingostrips and PIPstrips were normalized to input and the graph combines data from three independent Sphingostrip and four independent PIPstrip assays. Averages and standard error of the mean are shown.

**Figure 7:**
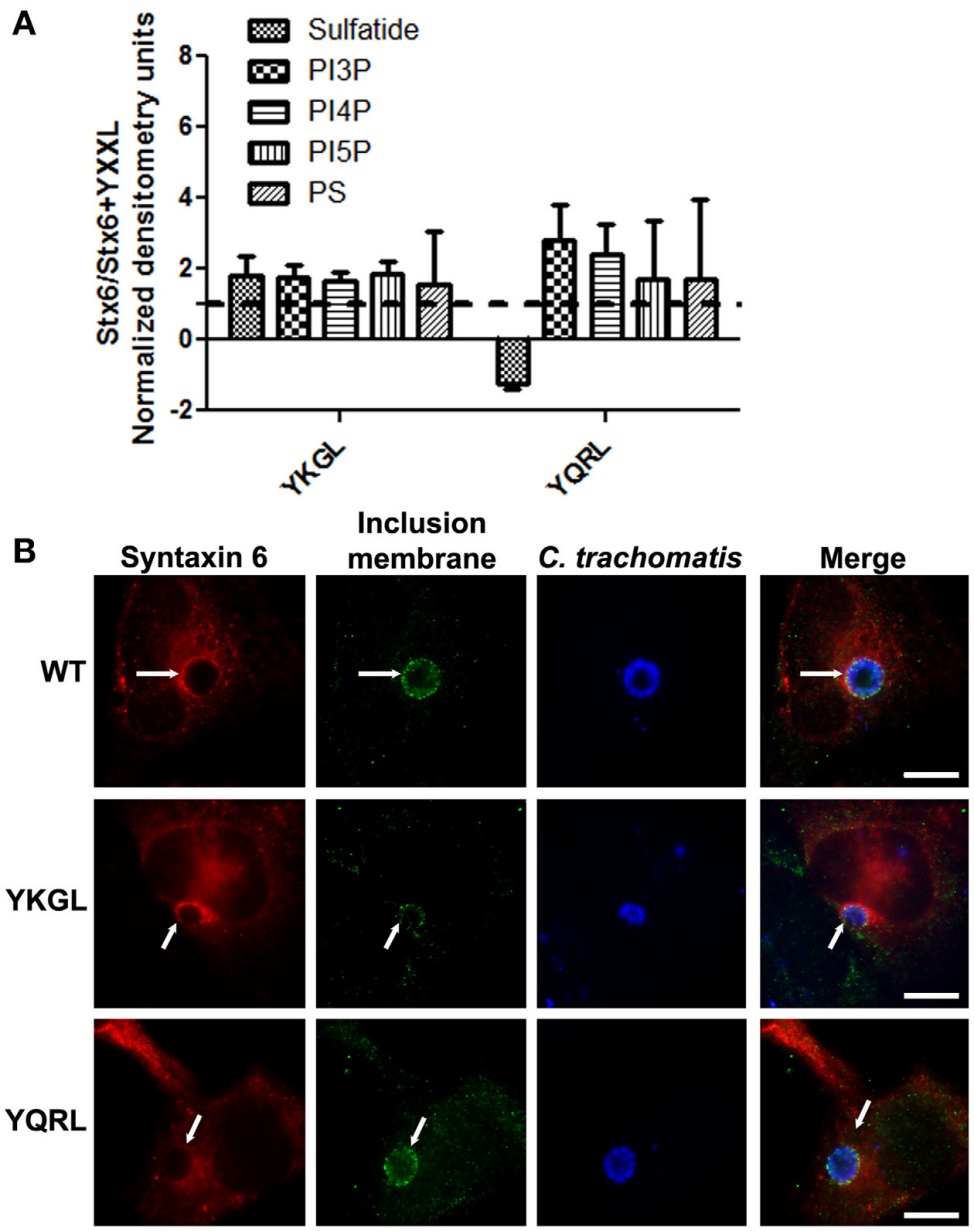
**Syntaxin 6 + YKGL or + YQRL protein-lipid interactions and localization to the chlamydial inclusion**. In **(A)**, 3XFLAG-syntaxin 6 wild-type (WT), 3XFLAG-syntaxin 6 + YKGL (YKGL), and 3XFLAG-syntaxin 6 +YQRL (YQRL) were immunoprecipitated from HeLa cells and incubated with Sphingostrips or PIPstrips. Fold increase in syntaxin 6-lipid binding was determined by normalization of densitometry to input. Graph depicts the fold changes in densitometry levels of WT compared to YKGL or YQRL, respectively. The graph combines three independent Sphingostrip and PIPstrip assays. Averages and standard error of the mean are shown. In **(B)**, HeLa cells were transfected with WT, YKGL, or YQRL constructs, infected and fixed in ethanol as described in Figure 3. The samples were processed for indirect immunofluorescence to detect the 3XFLAG (red), the inclusion membrane (green), or organisms (blue). Images are representative of two independent experiments. White arrows indicate colocalization with the inclusion; bars, 10 μm.

To examine if deletion of the YGRL signal sequence effects the ability of syntaxin 6 to bind to lipids, we purified 3XFLAG-syntaxin 6, 3XFLAG-syntaxin 6ΔYGRL, or 3XFLAG expressed from an empty vector from transfected HeLa cells. Equal amounts of protein were loaded onto either Echelon Sphingostrips (Figure 6A) or PIPStrips (Figure 6C) to screen for syntaxin 6-lipid interactions. Strips were developed by Western blot as described in Section Methods and Materials; protein inputs onto the strips are shown (Figures 6B,D). 3XFLAG-syntaxin 6 binds to sulfatide (sulfonated galactosylceramide), phosphatidylinositol 3-phosphate (PI3P), phosphatidylinositol 4-phosphate (PI4P), phosphatidylinositol 5-phosphate (PI5P), and phosphatidylserine (PS) (Figures 6A,C). Notably, PS and PI species have been purified from chlamydial organisms (Newhall, 1988), with PI4P demonstrated on the chlamydial inclusion membrane (Moorhead et al., 2010). Deletion of the YGRL from syntaxin 6, results in a decrease in lipid binding, with wild type syntaxin 6 having a 2.6–4.0 fold higher affinity for lipid species (Figure 6E). No background was obtained by strips treated with empty vector lysates, indicating the specificity of the interactions obtained (Figures 6A,C). We also examined the lipid affinity of 3XFLAG-syntaxin 6-YKGL and 3XFLAG-syntaxin 6-YQRL. The alteration of the YGRL signal sequence to either the YKGL or YQRL did not change the lipid binding profile of syntaxin 6, but did change the apparent affinity of syntaxin 6 for lipids similarly to deletion of the YGRL signal sequence (Figure 7A and Supplementary Figure 5). Interestingly, unlike the ΔYGRL mutant, both syntaxin 6 + YKGL and + YQRL localized to the chlamydial inclusion. The YGRL signal sequence confers affinity for specific lipids, but does not change the lipid binding profile of syntaxin 6. Additionally, an YXXL signal sequence is an absolute requirement of syntaxin 6 being trafficked to the inclusion.

## Discussion

Syntaxin 6 cycles between the trans-Golgi and the plasma membrane, and the YGRL signal sequence functions to return syntaxin 6 to the trans-Golgi from the plasma membrane. Without the YGRL signal sequence, syntaxin 6 remains at the plasma membrane or in vesicles between the plasma membrane and trans-Golgi (Watson and Pessin, 2000). We previously demonstrated that the YGRL signal sequence within syntaxin 6 was required for syntaxin 6 to localize to the chlamydial inclusion (Moore et al., 2011). In this study, we examined if other YXXL signal sequences could recognize the chlamydial inclusion, and if localization was being mediated by protein-protein or protein-lipid interactions. Notably, we demonstrate that only the YGRL signal sequence of syntaxin 6 redirects proteins that normally do not localize to the chlamydial inclusion (Figures 2, 3 and Supplemental Figure 1). The requirement of YGRL for syntaxin 6 localization is conserved across chlamydial species (Figure 4). Finally, deletion or alteration of the YGRL signal sequence in syntaxin 6 alters the affinity of syntaxin 6 to bind lipids (Figures 6, 7A and Supplemental Figure 5). Curiously, substitution of YGRL for YKGL or YQRL did not prevent syntaxin 6 from being properly trafficked to the chlamydial inclusion (Figure 7B). Collectively, these data indicate that an YXXL signal sequence plays a role in how some proteins are trafficked and recognized by the chlamydial inclusion.

It is currently unknown if syntaxin 6 is trafficked to the inclusion from the Golgi and/or from the plasma membrane. However, the BirA^*^-syntaxin 6ΔYGRL fusion protein never biotinylates the inclusion membrane (Figure 1A), indicating that without the signal sequence, syntaxin 6 is not trafficked to the inclusion membrane at all, not even transiently. This is consistent with how the YGRL signal sequence functions in the context of syntaxin 6 cycling between the trans-Golgi and the plasma membrane. Additionally, it builds upon data demonstrating that the inclusion interacts with other subcellular pathways in addition to a post-Golgi exocytic pathway (Hackstadt et al., 1996; Beatty, 2006; Moore et al., 2008; Ouellette and Carabeo, 2010). An interesting question that arises from these data is the idea that syntaxin 6 may be cycling between the inclusion membrane and the plasma membrane, similarly to how syntaxin 6 cycles between the trans-Golgi and plasma membrane. Studies within the lab are currently testing this possibility.

An important clue as to how YGRL functions in trafficking proteins to the chlamydial inclusion is the fact that the YGRL signal sequence recognizes inclusions formed by different species of *Chlamydia* (Figure 4). Chlamydiae modify the content of their inclusion membranes with Inc proteins (Scidmore et al., 1996; Rockey et al., 1997; Bannantine et al., 1998; Fields et al., 2003). These Inc proteins are quite diverse in nature, with very few being conserved across chlamydial species (Lutter et al., 2012). Likewise, the recruitment of host proteins, including Rab GTPases, Src kinases and some SNAREs, to the chlamydial inclusion is not conserved across chlamydial species (Rzomp et al., 2003, 2006; Moorhead et al., 2007; Lipinski et al., 2009; Capmany and Damiani, 2010; Mital and Hackstadt, 2011; Kabeiseman et al., 2013). We hypothesize that the YGRL signal sequence is responsible for trafficking syntaxin 6 *to* the inclusion, where interaction with protein binding partners, such as VAMP4 can occur. How the YGRL targets the chlamydial inclusion is still being investigated, but below we will discuss how protein-protein and protein-lipid interactions may mediate this process.

There is likely a common property or mechanism in how YGRL targets the trans-Golgi and the chlamydial inclusion membrane. Proteins containing the YKGL and YQRL signal sequences are trafficked appropriately based on the recognition of the signal sequence by the μ2 subunit of AP-1 and AP-2 containing complexes (Boll et al., 1996; Teuchert et al., 1999a,b; Cabrera et al., 2003). Additionally, the affinity of the YQRL signal sequence for AP-1 seems to be driven in part by interactions with phosphatidylinositol-3-phosphate (Rapoport et al., 1997). In the context of chlamydial infected cells, the recruitment of proteins containing the YGRL signal sequence is by an independent mechanism not shared with the recognition and recruitment of proteins containing the YQRL or YKGL signal sequences, as no wild type proteins containing an YQRL or YKGL are trafficked to the inclusion. In the case of syntaxin 6, deletion of the YGRL did not broadly redefine with which proteins syntaxin 6 interacted (Figure 5F). However, in Western blots of specific syntaxin 6 binding partners after immunoprecipitation, 3XFLAG-syntaxin 6ΔYGRL had an increased affinity for the 42 kDa isoform of syntaxin 5 (Figure 5C). Notably, the 42 kDa isoform of syntaxin 5 contains an ER-retention sequence, causing the protein to cycle between the ER and cis-Golgi (Hui et al., 1997). Therefore, these data support the notion that the change in affinity of syntaxin 6ΔYGRL for syntaxin 5 (42 kDa) is likely due to a change in subcellular localization of the mutant syntaxin 6 and not a biochemical alteration in protein-protein interactions. Previous studies reported that AP-1 also binds to syntaxin 6 (Bock et al., 1997). We attempted to examine whether AP-1 localizes to the inclusion or can bind to syntaxin 6ΔYGRL, but due to the poor reactivity of the commercially available antibody, we were unable to form any conclusions (data not shown). Therefore, we cannot definitively rule out that the YGRL signal sequence is being recognized by a common chlamydial protein or a recruited eukaryotic protein (such as AP-1). However, we previously demonstrated that knockdown of VAMP4 prevented localization of syntaxin 6 to chlamydial inclusions formed by *C. trachomatis* serovar L2 (Kabeiseman et al., 2013). Deletion of the YGRL from syntaxin 6 does not alter syntaxin 6-VAMP4 interactions as measured by immunoprecipitation (Figure 4B). The current data support the hypothesis that the YGRL signal sequence traffics syntaxin 6 to the chlamydial inclusion where it interacts with VAMP4 and is then retained on the chlamydial inclusion membrane. Current studies in the laboratory are testing this hypothesis.

To find a protein whose localization is conserved across chlamydial species was unusual given the diversity of chlamydial Inc proteins and host proteins which localize to the inclusion. That the protein also contains a signal sequence that recognizes the chlamydial inclusion is intriguing. A common denominator amongst all of the Chlamydiae is the acquisition of host-derived lipids, including sphingomyelin and cholesterol (Hackstadt et al., 1996; Wolf and Hackstadt, 2001; Carabeo et al., 2003). Given that the YGRL signal sequence recognizes all of the inclusions formed by various species of *Chlamydia*, it may be recognizing common features on the chlamydial inclusion membranes, which may be a lipid component. This hypothesis is supported by the fact that syntaxin 6ΔYGRL has a lower affinity for sulfatide, phosphatidylinositol 3-phosphate (PI3P), phosphatidylinositol 4-phosphate (PI4P), phosphatidylinositol 5-phosphate (PI5P), and phosphatidylserine (PS) than wild type syntaxin 6 (Figure 6). PI4P, PI5P, and PS are incorporated into chlamydial organisms, whereas sulfatide and PI3P are not (Newhall, 1988; Wylie et al., 1997; Moorhead et al., 2010). However, syntaxin 6 +YKGL and syntaxin 6 +YQRL show similar lower affinities for the lipids described above and yet are trafficked to the chlamydial inclusion (Figure 7). While these data do not necessarily negate the possibility of a lipid component recognizing the YGRL signal sequence at the chlamydial inclusion, it does indicate the tyrosine and leucine amino acids are important for this recognition/interaction. Moreover, there appears to be some measure of flexibility in the YXXL signal sequence that results in syntaxin 6 being targeted to the inclusion. This is unique, as additions of YKGL and YQRL to syntaxin 4 did not result in that protein being trafficked to the inclusion. One explanation is that mutation of the YGRL in syntaxin 6 to YKGL or YQRL does not appreciably alter subcellular localization and in which trafficking pathway syntaxin 6 functions. Whereas addition of any YXXL signal sequence to syntaxin 4 appreciably alters subcellular localization, but only the YGRL results in positioning the protein within a trafficking pathway that interacts with the chlamydial inclusion. Further, deletion of the YGRL in syntaxin 6 markedly alters subcellular localization and presumably the cognate trafficking pathway, resulting in lack of targeting of the mutant protein to the inclusion.

The recognition of the YGRL signal sequence by the inclusion may be indicative of how syntaxin 6 is trafficked and how it functions at the inclusion, but it may also be a marker for how *Chlamydia* remodel their pathogen-specified organelle. Vacuoles containing *Legionella* and *Brucella* escape the endocytic pathway by subverting autophagy machinery and associating with endoplasmic reticulum (ER) membranes (Swanson and Isberg, 1995; Pizarro-Cerda et al., 1998a,b). Additionally, both pathogen-containing vacuoles are recognized by the classical ER-retention signal, KDEL (Kagan and Roy, 2002; Salcedo et al., 2008). However, maturation of autophagosomes does not include the maintenance of ER-markers, such as GFP-KDEL (Campbell-Valois et al., 2012). Therefore, the fact that *Brucella* or *Legionella*-containing vacuoles retain ER markers for extended periods of time suggests a pathogen-specified alteration to normal cellular processes for the purpose of establishing and maintaining their intracellular niche (Campbell-Valois et al., 2012). Further, the ability of *Legionella* and *Brucella* to retain KDEL-containing proteins strongly suggests that they are retaining the KDEL “receptor,” which is a protein(s) (Cabrera et al., 2003; Raykhel et al., 2007) on their vacuolar membranes. In the case of the YGRL signal sequence and the recognition of the chlamydial inclusion, our current data suggest that the YGRL may be binding to a “receptor” but it is not clear whether this “receptor” is lipid or protein in nature.

## Concluding remarks

For the first time, we identify a eukaryotic signal sequence that can target the chlamydial inclusion. Further, the YGRL signal sequence recognizes inclusions formed by multiple species of *Chlamydia*, indicating that it is interacting with a conserved component that is integral to inclusion integrity or composition. The YQRL, YKGL, and YGRL signal sequences all function to return proteins to the trans-Golgi, yet only the YGRL, when added to proteins that normally do not traffic to the chlamydial inclusion, is recognized by the chlamydial inclusion. Uniquely, in the context of syntaxin 6 trafficking, YKGL or YQRL can substitute for YGRL in localizing it to the chlamydial inclusion. These data highlight the importance of an YXXL signal sequence in the trafficking of syntaxin 6 to the chlamydial inclusion. Understanding the interactions that occur at the chlamydial inclusion, including how proteins/lipids are targeted to the inclusion, is fundamental to understanding how these pathogens exact silent infections.

### Conflict of interest statement

The authors declare that the research was conducted in the absence of any commercial or financial relationships that could be construed as a potential conflict of interest.
