# Supplementary material for: The eukaryotic signal sequence, YGRL, targets the chlamydial inclusion
Source: Front Cell Infect Microbiol. 2014 Sep 11;4:129. doi: 10.3389/fcimb.2014.00129 (PMC4161167; doi:10.3389/fcimb.2014.00129)
Supplement: Supplementary file 1 [file DataSheet1.DOCX]

**Protein identification by mass spectrometry analysis**

The excised band containing the protein was in-gel digested using trypsin sequencing grade (Promega, Madison Wi) and nanoRPLC in-line desalted as described previously (Mo B *et. al.*; 2006,2008). Peptide analysis was performed using nanoLC-ESI-MS/MS. The peptides were in-line desalted and concentrated with an RP-Trap column Symmetry C18, 5 µm, 180 µm (id) X 20 mm nanoAcquity UPLC column (Waters Corp., Milford, MA), and separated using a BEH130 C18 RP, 1.7  µm , 100  µm (id) X 100 mm nanoAcquity UPLC column (Waters Corp., Milford, MA). The standard gradient used was: 0-2 min, 3% B isocratic; 2-40 min, 3-80 % B linear. Mobile phase A was water/formic acid (99.9:0.1, v/v), and phase B was acetonitrile/formic acid (99.9:0.1 v/v). The solvent flow rate was 400 nl/min. The separation was performed using nanoAcquity Ultra Performance LC (nanoAcquity UPLC (Waters Corp., Milford, MA). The eluted ions were analyzed by one full precursor MS scan (400-1500 m/z) followed by four MS/MS scans of the most abundant ions detected in the precursor MS scan while operating under dynamic exclusion or direct data acquisition system Spectra obtained in the positive ion mode with nano ESI Q-Tof Synapt G1 HDMS mass spectrometer (Waters, Milford, MA) were deconvoluted, and analyzed using the MassLynx software 4.1 (Micromass, UK). A peak list (PKL format) was generated to identify +1 or multiple charged precursor ions from the mass spectrometry data file. The instrument was calibrated in MS/MS mode using 100 fmole of (Glu^1^)-Fibrinopeptide B human with a root mean square residual of 3.495 e^-3^ amu or 7.722 e^0^ ppm. Parent mass (MS) and fragment mass (MS/MS) peak ranges were 400-2000 Da and 65-2000 Da, respectively.

Mascot server v2.4 ([www.matrix-science.com](http://www.matrix-science.com), UK) in MS/MS ion search mode (local licenses) was applied to conduct peptide matches (peptide masses and sequence tags) and protein searches against NCBInr v20140122 (35149712 sequences; 12374887350 residues) using all entries as well as taxonomy filter for Human (Homo sapiens) (272342 sequences). The following parameters were set for the search: carbamidomethyl (C) on cysteine was set as fixed; variable modifications included asparagine and glutamine deamidation and methionine oxidation. Only one missed cleavage was allowed; monoisotopic masses were counted; the precursor peptide mass tolerance was set at 1 Da; fragment mass tolerance was 0.3 Da and the ion score or expected cut-off was set at 5. The MS/MS spectra were searched with MASCOT using a 95% confidence interval (C.I. %) threshold (p<0.05), with which minimum score of 48 was used for peptide identification. The protein redundancy that appeared at the database under different gi and accession numbers were limited to Human. All of the proteins identified in the current study were found these domains.

Mo B, Callegari E, Telefont M, Renner KJ, 2006. “Proteomics analysis of ventromedial nucleus of the hypothalamus (pars lateralis) in the female rat”. *Proteomics* 6:6066-6074 (PubMed:17051637).

Mo B, Callegari E, Telefont M, Renner KJ, 2008. “Estrogen regulation of proteins in the rat ventromedial nucleus of the hypothalamus”. *J. Proteome Research* 7(11): 5040-8.
