# Supplementary material for: The eukaryotic signal sequence, YGRL, targets the chlamydial inclusion
Source: Front Cell Infect Microbiol. 2014 Sep 11;4:129. doi: 10.3389/fcimb.2014.00129 (PMC4161167; doi:10.3389/fcimb.2014.00129)
Supplement: Supplementary file 2 [file DataSheet2.DOCX]

**Supplemental Figure 1. Targeting furin and Tgn38 to the chlamydial inclusion.** HeLa cells were transfected with control construct 3XFLAG vector (C) and 3XFLAG-furin or 3XFLAG-Tgn38 and their respective YGRL mutant constructs (A & B). After infection with *C. trachomatis* serovar L2 (*Ct*) (A) or *C. burnetii* (*Cb*) (B), the cells were fixed in ethanol and processed for indirect immunofluorescence assay to detect 3XFLAG (red), *C. trachomatis* (green, A & C) or *C. burnetii* (green, B). Each image is representative of at least two independent experiments. White stars designate the chlamydial inclusion, white caret designates the *C. burnetii* vacuole and white arrows denote colocalization of furin YGRL and Tgn38 YGRL with the chlamydial inclusion. Bars, 10 μm.

**Supplemental Figure 2**. HeLa cells were transfected with 100 ng of 3XFLAG-furinΔYKGL or 3XFLAG-Tgn38ΔYQRL as described in methods. Then, cells were infected with *C. trachomatis* serovar L2 for 16-18 hours prior to fixation and processing for indirect immunofluorescence. 3XFLAG constructs were detected with an mouse anti-FLAG M2 antibody (red) and an rabbit anti-*Chlamydia* organism antibody (green), followed by appropriate secondary antibodies. Images are representative of a minimum of 3 independent experiments. Bars, 10µm.

**Supplemental Figure 3.** HeLa cells were infected with avirulent *Coxiella burnetii* Nine mile Phase II organisms for 72 hours, then transfected with 3XFLAG-Furin_YGRL as described in methods. 24 hours later, cells were fixed and processed for indirect immunofluourescence. 3XFLAG constructs were detected with an anti-FLAG M2 antibody (red) and an guinea pig anti-*Coxiella* organism antibody (green), followed by the appropriate secondary antibodies. Images shown are representative from 2 independent experiments, with one representative image from each experiment. Bars, 10µm.

**Supplemental Figure 4.** HeLa cells were infected with *C. trachomatis* serovar L2 for 16-18 hours prior to fixation in 4% paraformaldehyde. They were processed for indirect immunofluorescence with the indicated primary and appropriate secondary antibodies. White starts indicate the chlamydial inclusion. Images shown are representative from 2 independent experiments. Bars, 10µm.

**Supplemental Figure 5.** **Effect of YKGL and YQRL signal sequences on the lipid affinity of syntaxin 6.** 3XFLAG-syntaxin 6 (WT), 3XFLAG-syntaxin 6-YKGL (YKGL) or 3XFLAG-syntaxin 6-YQRL (YQRL) were immunopreciptated from HeLa cells and 2.8 µg of eluate were incubated with PIPstrips. Protein-lipid interactions phosphatidylinositol 3-phosphate (PI3P), phosphatidylinositol 4-phosphate (PI4P), phosphatidylinositol 5-phosphate (PI5P), and phosphatidylserine (PS) were detected by blotting with anti-3XFLAG. The blank is illuminated for orientation purposes. Protein input (1 µg/well) for the PIP strips was analyzed by blotting with anti-FLAG M2 antibody. The images are representative of three independent experiments. Raw densitometry values were determined using Image Studio v 2.0 and are provided for each positive reaction on the PIPstrip and for the bands on the Western blot.
