# Supplementary figures and images for: The eukaryotic signal sequence, YGRL, targets the chlamydial inclusion
Source: Front Cell Infect Microbiol. 2014 Sep 11;4:129. doi: 10.3389/fcimb.2014.00129 (PMC4161167; doi:10.3389/fcimb.2014.00129)

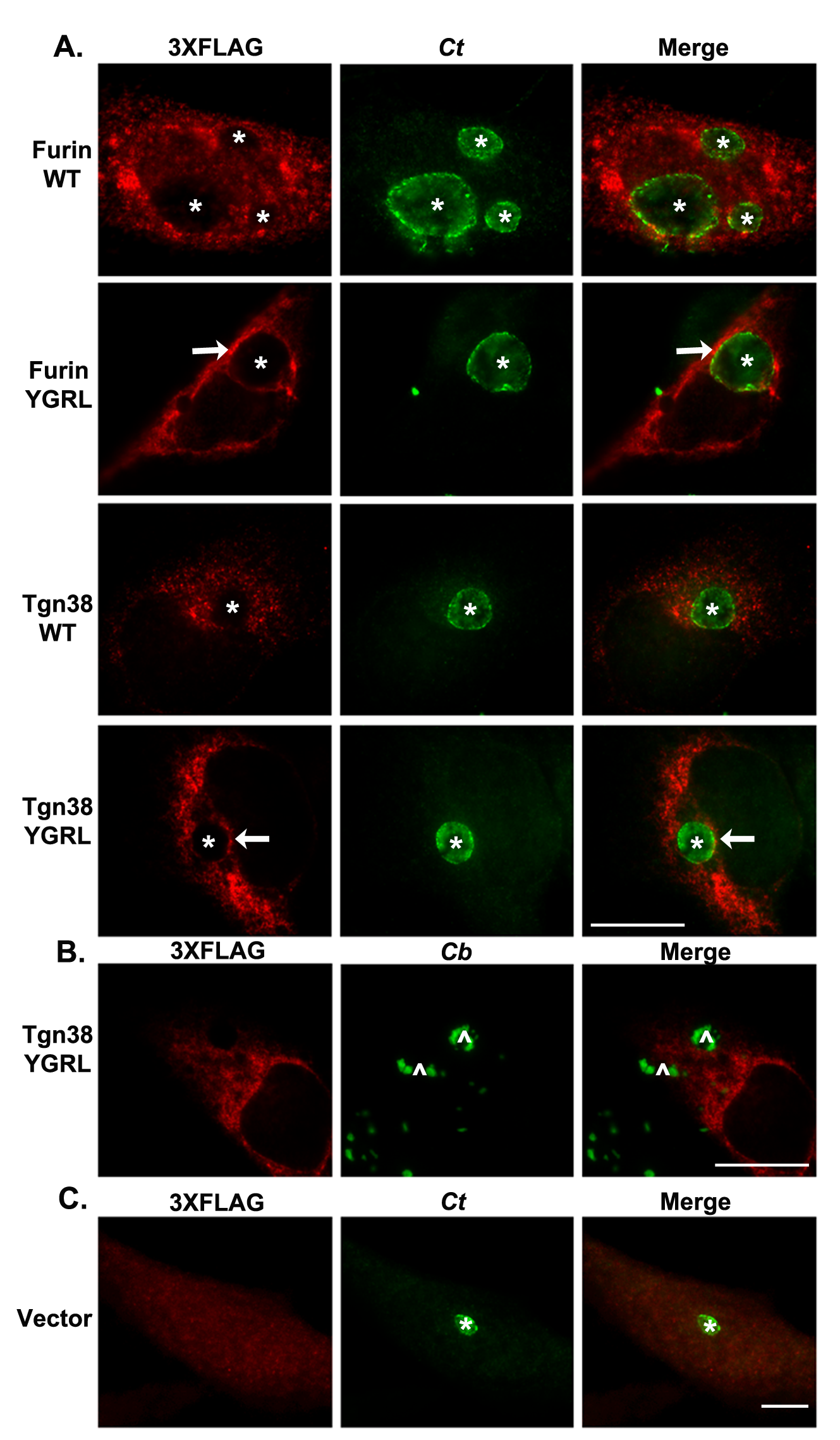

Supplement: Supplementary file 3 [file Image1.TIF]

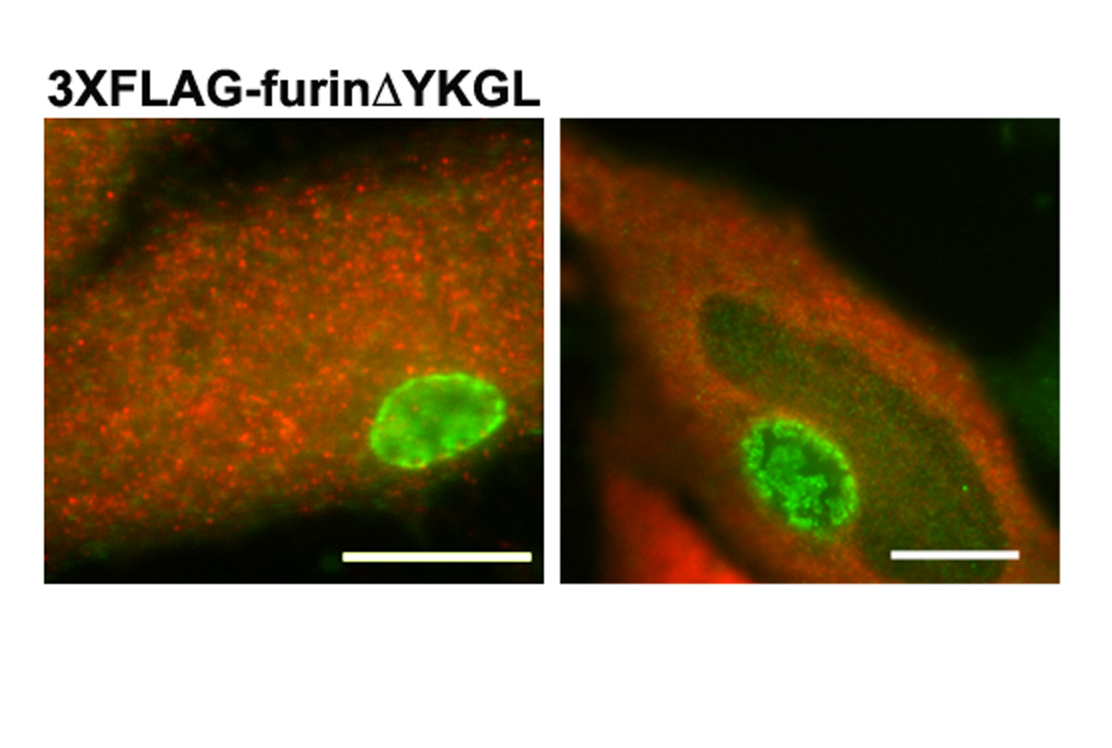

Supplement: Supplementary file 4 [file Image2.TIF]

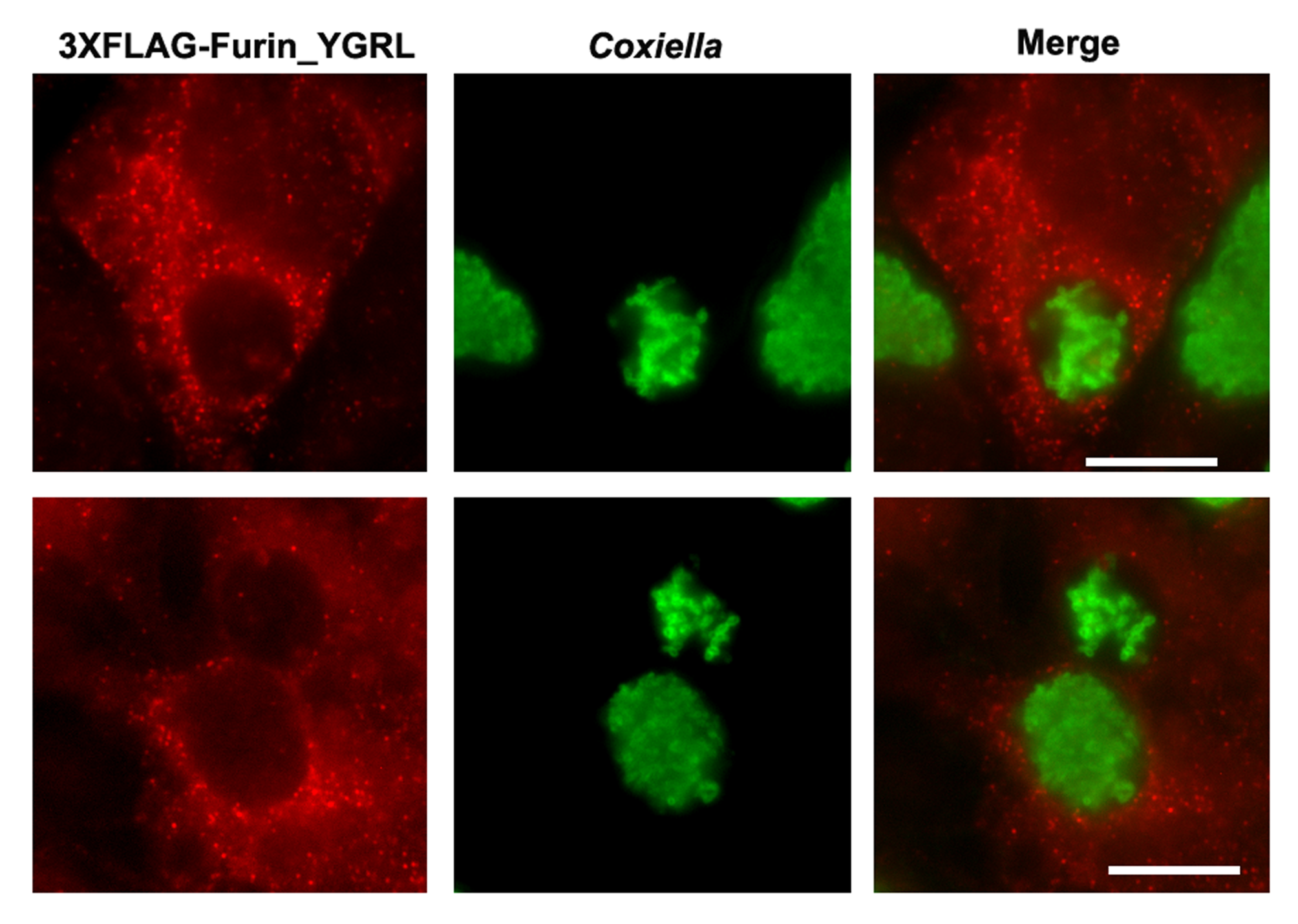

Supplement: Supplementary file 5 [file Image3.TIF]

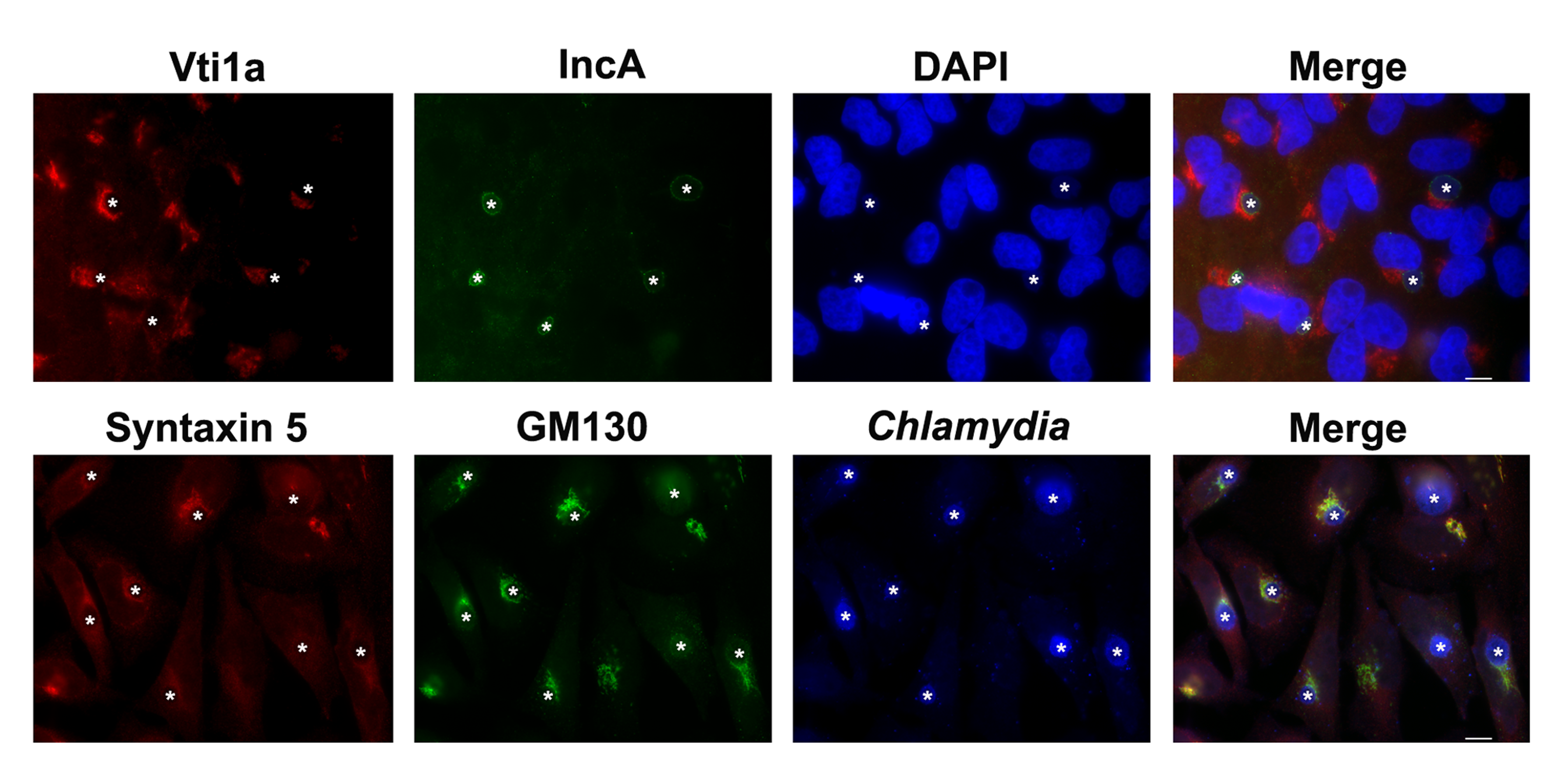

Supplement: Supplementary file 6 [file Image4.TIF]

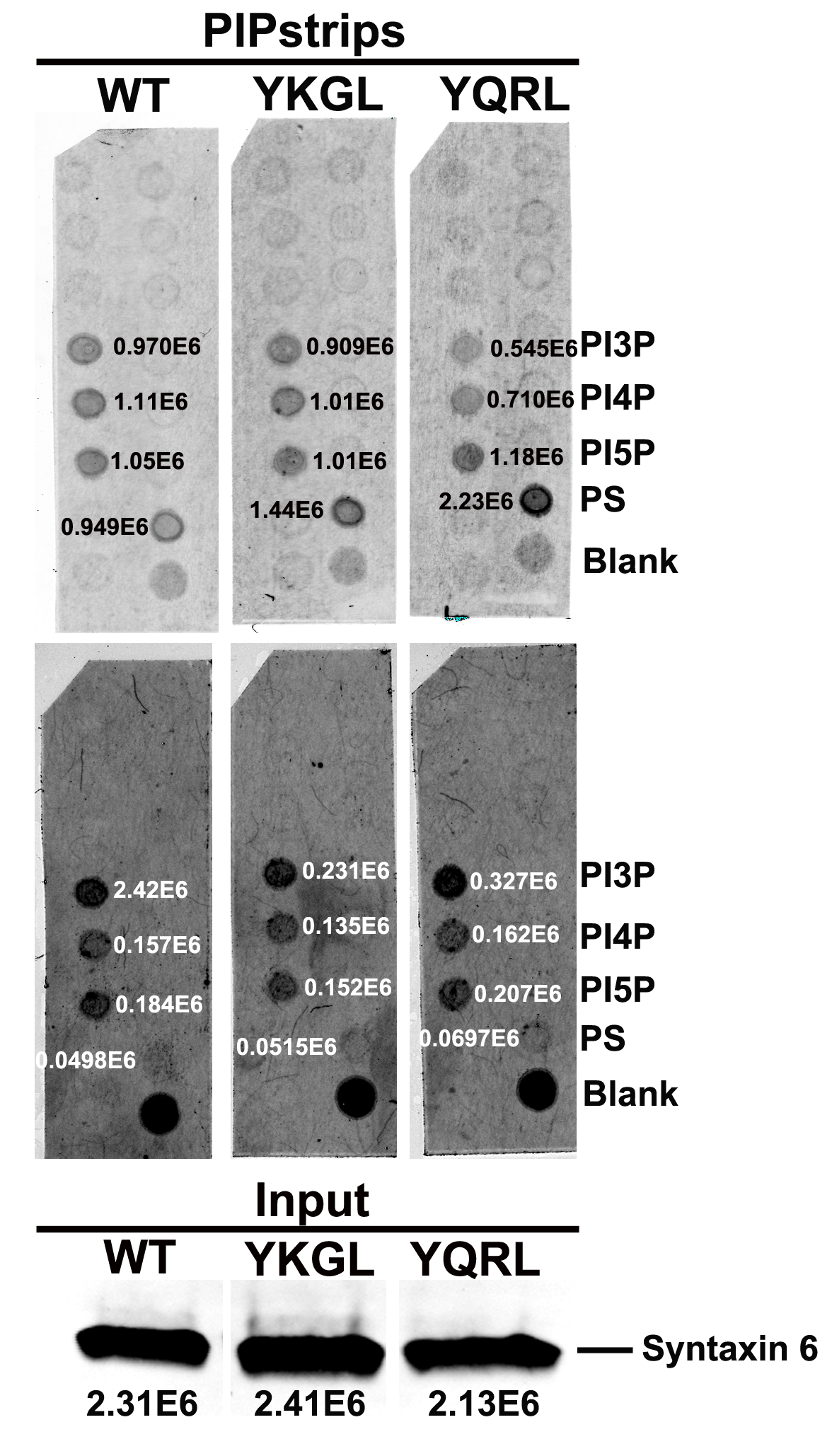

Supplement: Supplementary file 7 [file Image5.TIF]
